# SPECT/CT in the Evaluation of Suspected Skeletal Pathology

**DOI:** 10.3390/tomography7040050

**Published:** 2021-10-11

**Authors:** Bhasker Rao Koppula, Kathryn A. Morton, Ragheed Al-Dulaimi, Gabriel C. Fine, Nikolas M. Damme, Richard K. J. Brown

**Affiliations:** Department of Radiology and Imaging Sciences, University of Utah, Salt Lake City, UT 84132, USA; Kathryn.Morton@hsc.utah.edu (K.A.M.); Ragheed.Al-dulaimi@hsc.utah.edu (R.A.-D.); Gabriel.Fine@hsc.utah.edu (G.C.F.); Nik.Damme@hsc.utah.edu (N.M.D.); rkjbrown@hsc.utah.edu (R.K.J.B.)

**Keywords:** computed tomography, single photon emission computed tomography, fusion imaging, hybrid imaging, scintigraphy, musculoskeletal pathology

## Abstract

Dedicated multi-slice single-photon emission computed tomography/computed tomography (SPECT/CT) cameras have become widely available and are becoming a mainstay of clinical practice. The integration of SPECT and CT allow for precise anatomic location of scintigraphic findings. Fusion imaging with SPECT/CT can improve both sensitivity and specificity by reducing equivocal interpretation in comparison to planar scintigraphy or SPECT alone. This review article addresses the technique, basic science principles, and applications of integrated SPECT/CT in the evaluation of musculoskeletal pathology.

## 1. Introduction

**Figure 1:**
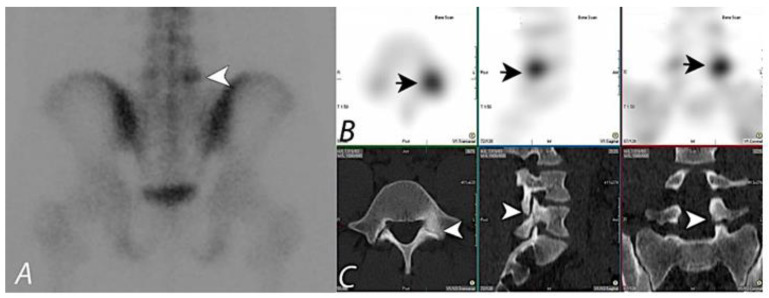
^99m^Tc MDP bone scan planar (spot) view image (**A**) of pelvis on a young patient presenting with chronic left sided low back pain, showing focal uptake (black arrowhead) in the lower lumbar region on the left side. SPECT images in axial, sagittal, and coronal planes (**B**) localizes the activity to posterior aspect of L5 vertebral body on the left side (Short black arrows). Correlative CT images in axial, sagittal, and coronal planes (**C**) demonstrate a bony defect in the isthmus of the left L5 level pars intraarticularis (white arrowheads), consistent with a monolateral spondylolysis. Taking into consideration the pars defect on anatomic imaging at this level and the focal activity on the bone scan, it can be assumed that the activity most likely correlates with the anatomic abnormality. Lack of co-registration of anatomic and functional information on this set of images prevents definitive interpretation, and hybrid SPECT/CT with fused information can significantly improve diagnostic confidence.

A variety of conditions, including degenerative disease, neoplastic processes (both benign and malignant), infection, inflammation, metabolic disease, and trauma may affect the musculoskeletal system. Bisphosphonate radiopharmaceuticals, which are analogs of calcium pyrophosphate, are deposited with a greater affinity at the sites of altered bone turnover and remodeling at sites of increased osteoblastic activity compared to normal bone. Bone scintigraphy with these agents can detect these functional/metabolic changes long before the morphologic changes become apparent on conventional anatomic imaging modalities [1,2,3,4,5,6,7,8,9,10,11]. In addition, whole-body scintigraphy has a unique and significant advantage over anatomic imaging modalities, in that it can assess the integrity of the entire skeleton. Planar bone scintigraphy (bone scan) with a ^99m^Tc bisphosphonate agent is a highly sensitive imaging modality for depiction of early functional derangements of the bone, resulting in increased osteoblastic remodeling. However, specificity is lacking in that many conditions varying in severity and clinical significance may result in osteoblastic remodeling. Furthermore, planar bone scintigraphy lacks 3-dimensional contrast in the same way that an AP radiograph of the abdomen lacks 3-dimensional contrast when compared to abdominal CT scan. The addition of single photon emission computed tomography (SPECT) provides the 3-dimensional contrast lacking in planar imaging, but this is still limited in that the precise area of uptake and the morphological characteristics of the bone that provide information as to the cause of the increased osteoblastic remodeling are lacking. Improved correlative information may be provided by side-to-side comparisons between SPECT and CT scans or plain radiographs, but localization is often only approximate (Figure 1). Combined single-photon emission computed tomography/computed tomography (SPECT/CT) overcomes this limitation by combining the functional information obtained by SPECT with precise localization and morphologic characterization of CT at the site of uptake. The diagnostic value and complimentary nature of combined functional and anatomical imaging is increasingly recognized [12].

Image fusion software allows co-registration of information acquired on different instruments and imaging modalities, usually at different time points, and has been shown to be relatively accurate with rigid structures such as the brain, as well as with extremities [13]. However, fusion imaging has been shown to be challenging in the evaluation of the chest and abdomen. Various factors such as patient position during imaging, patient motion, and inherent motion of internal organs have proven to be difficult in achieving accurate fusion. In the context of evaluation of skeletal pathology, particularly spine, apart from patient factors, technical factors such as curvature and shape of the imaging tables have been shown to lead to different positioning of the spine on different scanners, limiting accurate fusion of anatomic and functional information [14,15,16]. In clinical practice, usage of image fusion software was found to be time consuming and, sometimes, technically difficult to achieve, leading to delayed diagnosis.

**Figure 2:**
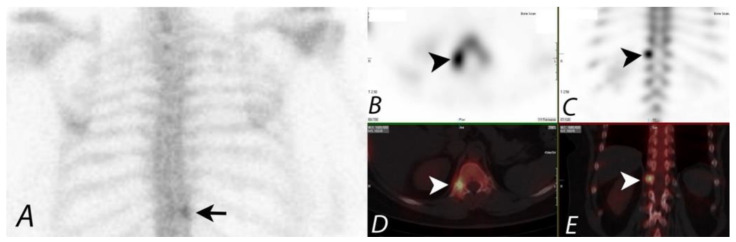
Patient presenting with chronic right sided mid back pain. ^99m^Tc MDP bone scan planar posterior spot view (**A**) of chest demonstrates a focal area of uptake (black arrow) in the lower thoracic region in paramedian location on the right side. SPECT images in axial and coronal planes (**B**,**C**) confirm the focal uptake localizing to the posterior element (black arrowheads), though it is unclear if this represents activity within the posterior aspect of the vertebral body, pedicle, or within the facet joints. Fused SPECT/CT images (**D**,**E**) in axial and coronal planes accurately localize the accumulation of the tracer activity to the costovertebral junction on the right side (white arrowheads), likely due to degenerative change.

Acquisition of functional and morphologic information on integrated positron emission tomography (PET)/Computed tomography systems has been shown to increase the diagnostic performance of Positron emission tomography (PET) alone, leading to widespread adoption of integrated hybrid imaging systems [17]. Although integrated SPECT/CT systems were explored by Hasegawa et al. in the early 1990s, only with the success of PET/CT systems was there significant commercial interest in developing a similar hybrid system for SPECT, and SPECT/CT has shown great promise in improving the diagnostic yield of planar imaging or SPECT alone by improving the specificity and accurate anatomic localization of lesions [18]. This new era of multimodality imaging with hybrid technology is broadening the scope of nuclear medicine [19] (Figure 2).

Hasegawa et al. [20] have devised an integrated system combining hardware components capable of performing both SPECT and CT and developed algorithms for SPECT attenuation correction using CT images. The first commercial SPECT/CT system, called the Hawk-Eye system, was developed by General Electric and was introduced in 1999. The system mounted a low dose x-ray tube on the same gantry as a dual-detector variable angle gamma camera [21,22]. CT images were used to provide a high-quality attenuation map for the SPECT images and provided fair anatomic images with the benefit of lower radiation to the patient compared with those of conventional CT [20,21,22,23]. Due to rapid technical developments and advancements, the current SPECT/CT systems offer diagnostic quality CT systems with options of multi-slice CT technology, typically with 2-, 6-, or 16-slice options. Of note, algorithms to reduce metal-reduction artifacts, which may be important in evaluating joint prosthetic complications, typically require a 16-slice CT scanner. Additional software algorithms that constrain the SPECT image to the attenuation characteristics of bone by using the CT as a frame-of-reference for reconstruction, for example, xSPECT Bone^®^ (Siemens Healthineers, (Erlangen, Germany)) offer further improvements in image quality over conventional SPECT/CT. Determination of standardized uptake values (SUVs) on SPECT/CT images is also now available as a software option. New detector technology, for example, cadmium-zinc-telluride (CZT), offers enhanced resolution, reduced scatter, and greater sensitivity compared to conventional sodium-iodine (NaI) detector technology.

## 2. Oncologic Applications of Bone SPECT/CT

**Figure 3:**
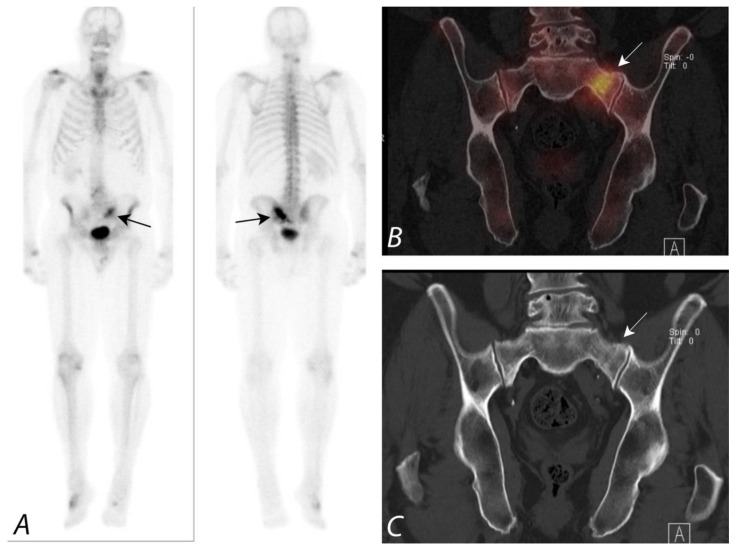
Anterior and posterior whole body ^99m^Tc MDP bone scan planar images (**A**) from a Tc-99m MDP bone scan in a 68-year-old male with prostate cancer and new pelvic pain demonstrate focal uptake (black arrows) localizing to the left sacral region. Coronal CT and fused SPECT/CT images (**B**,**C**) demonstrate the uptake localizing to a vertical insufficiency fracture of the left sacral ala, which demonstrates osteoblastic activity, confirming a healing insufficiency fracture and not metastatic disease.

Whole-body bone scintigraphy is the standard procedure in the setting of evaluation of skeletal metastatic disease, as the presence or absence of skeletal involvement is a critical factor in management and prognosis in cancer patients. For further characterization and evaluation of the abnormalities identified on the whole-body imaging, additional focused planar/spot views of the area(s) of interest can be acquired. In case of a negative scan, no further imaging is needed. However, differentiating between benign and malignant bone disease may be difficult with planar scintigraphy alone. The addition of SPECT alone is reported to improve sensitivity and diagnostic accuracy through improved tracer localization, particularly in the vertebral column [23,24,25]. In addition, coexisting spinal pathology, such as severe degenerative disease and compression fractures, can make diagnosis of malignant infiltration or disease difficult [26]. As a general rule, uptake in the vertebral body or the pedicle alone is more likely to represent malignant disease than a pattern of uptake isolated to the vertebral body periphery or at the facet joints [27,28]. However, SPECT alone is often insufficient for precise localization and characterization of bone lesions, and correlation with anatomical imaging is often required to improve specificity (Figure 3). Because the diagnosis of bone metastases has significant prognostic and treatment implications, such as and the need for additional or intensified treatment, the accurate differentiation between benign and malignant lesions is of paramount importance [29,30,31].

**Figure 4:**
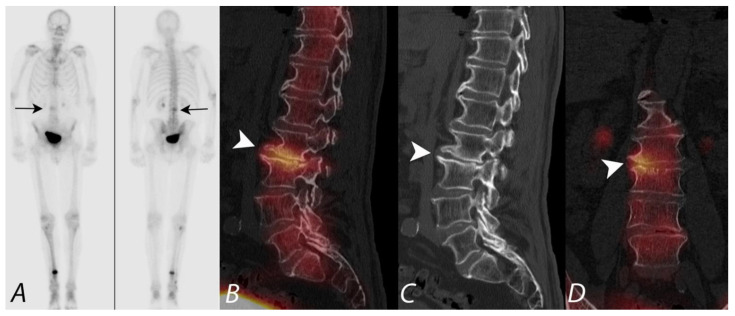
Anterior and posterior planar images (**A**) from a ^99m^Tc MDP bone scan on a patient with prostate cancer being evaluated for metastatic disease, with focal uptake along the right aspect of lumbar spine (black arrows). Additional focus of uptake involving the distal right tibia is related to a known fracture involving this region. No additional foci of uptake concerning metastatic disease were identified. Sagittal CT image and fused SPECT/CT images in sagittal and coronal projections (**B**–**D**) localize the activity to the right aspect of the disc space between L2 and L3, correlating with disc space narrowing and osteophytosis (white arrowhead). Findings are consistent with uptake related to degenerative disc disease and not related to metastatic disease.

Multiple studies have reported increased viewer confidence in interpretation by using the fusion of SPECT data with CT data when compared to SPECT alone or by side-by-side interpretation of anatomic imaging information from CT and scintigraphic data [32,33]. A comparative study of planar scintigraphy, SPECT, and SPECT-guided CT, digitally fused with 64-slice MSCT images in 37 patients with 42 focal bone lesions of the axial skeleton, reported the fused images to significantly increase diagnostic specificity [34] A specific diagnosis was made with planar scintigraphy in 64% of cases, SPECT in 86%, and SPECT fused with CT in all cases. The addition of SPECT/CT greatly improves the diagnostic value of Tc-99m bisphosphonate bone scans over that of planar imaging only (Figure 4).

**Figure 5:**
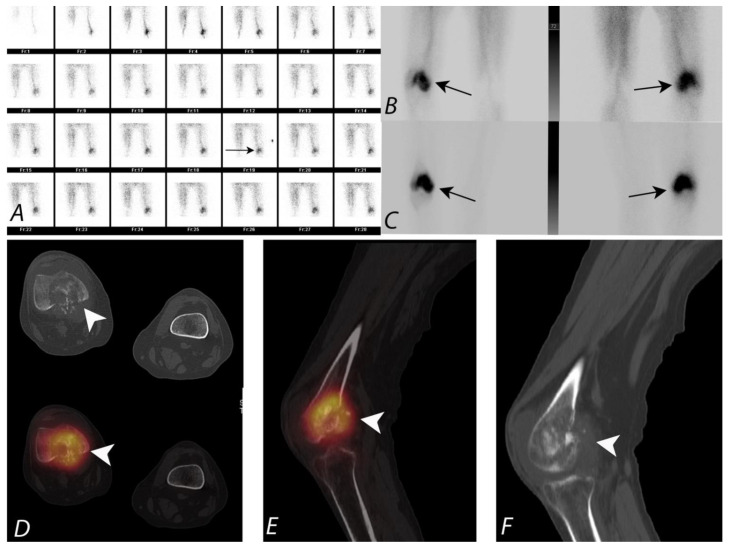
Twenty-year-old male patient presenting with right knee pain. Plain radiograph demonstrated a lesion suspicious for osteosarcoma involving the distal femoral shaft (Not shown). Patient underwent three phase ^99m^Tc MDP bone scan as a part of staging. Blood flow images (**A**) in posterior projection demonstrated asymmetrically increased blood flow to the right distal femoral region (black arrow). Blood pool and delayed images (**B**,**C**) also demonstrated increased pool activity and delayed accumulation of activity, correlating with the region of increased vascularity noted on blood flow images (black arrows). Axial CT and fused SPECT/CT (**D**), sagittal fused SPECT/CT (**E**) and sagittal CT images (**F**) images localize the activity to right distal femoral condyle predominantly lytic lesion associated with osteoid matrix. There is posterior cortical destruction, associated with soft tissue extension of the tumor posteriorly, which also demonstrates activity within (white arrowheads). Small knee joint effusion is also seen.

Several studies have demonstrated the benefits of using integrated SPECT/CT compared with SPECT in differentiating benign bone lesions from malignant bone lesions on ^99m^Tc bisphosphonate bone scans. In a prospective study by Horger et al. [34], SPECT/CT correctly classified 85% of indeterminate scintigraphic bone lesions compared with only 36% using SPECT alone. In a retrospective study by Romer et al. [35], 52 lesions in 44 patients were rated as indeterminate on SPECT alone. SPECT/CT was able to clarify 92% of these indeterminate lesions as benign findings, and 8% of the lesions remained indeterminate. In another study by Barwick et al., a definitive diagnosis was made on SPECT/CT in 87% (83/95) of indeterminate foci in 48 patients compared with only 30% using SPECT alone. SPECT/CT also identified additional sites of metastases in 21% (10/48) of patients [36]. In a retrospective study by Helyar et al., involving 40 consecutive patients, 61% of lesions were identified as indeterminate after evaluation of planar and SPECT images [37]. However, on SPECT/CT scans, only 8% of lesions were rated as equivocal, with the remainder of the lesions clearly identified either as benign or malignant lesions, thus improving diagnostic confidence [37,38] (Figure 5).

Small lesions can be missed if low-dose CT protocols are used for the CT portion of SPECT/CT imaging. Multiple studies have reported no significant difference in definitive characterization of equivocal lesions (86–92%) despite the different CT systems [36,37,38]. If the patient has had a recent diagnostic CT, it needs to be determined whether side-by-side comparison of this with the SPECT is as adequate as SPECT/CT. The potential of diagnostic contrast-enhanced SPECT-CT imaging has not yet been explored fully and raises the possibility of streamlined pathways of investigation in selected patients such as those with locally advanced breast cancer.

## 3. Non-Oncologic Applications of Bone SPECT/CT

**Figure 6:**
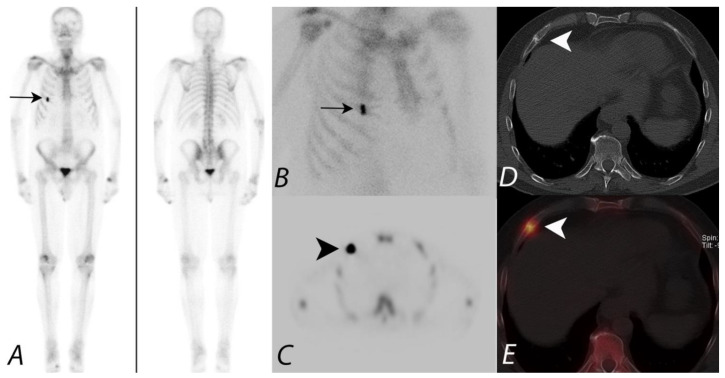
Patient presenting with prostate cancer underwent ^99m^Tc MDP bone scan to evaluate for metastatic disease. Whole body image in anterior projection (**A**) showing focal increased tracer uptake along the anterior aspect of the right sided 5th rib, confirmed on planar oblique image (**B**) (black arrows). SPECT image (**C**) demonstrates focal uptake along the 5th rib anterior aspect (black arrowhead). However, it is unclear if this focus represents a solitary metastatic disease versus another process, such as trauma. Axial CT (**D**) at the same level provides anatomic information showing a healing fracture (white arrowhead). Fused SPECT/CT image (**E**) accurately registers the focal activity (white arrowhead) identified on the planar image and SPECT images to the healing fracture on CT image—a great advantage of the hybrid SPECT/CT imaging combining the functional information with anatomic information.

The evidence base for the use of SPECT/CT in the evaluation of benign musculoskeletal pathology is rapidly emerging. In a prospective study by Evan-Sapir et al., involving 76 non-oncological patients with non-specific findings on planar imaging, SPECT/CT was of added clinical value in 89% of patients. Characterizing scintigraphic lesions by their morphologic appearance, SPECT/CT reached a final diagnosis in 59% of patients, obviating the need for further imaging [38]. In a study by Linke et al. [39] involving 71 patients without cancer who had extremity pain and underwent three-phase ^99m^Tc bisphosphonate bone scintigraphy and SPECT/CT of either the upper (n = 20) or the lower (n = 51) extremities. Four patients had no abnormal bone activity or CT abnormality in the extremities [39]. Among 34 lesions classified as osteoarthritis on planar and SPECT images, 7 were reclassified as fractures and one as a benign tumor with SPECT/CT. Of 15 lesions initially classified as osteomyelitis, 4 were diagnosed as osteoarthritis, 4 as fracture, and 1 as inflammation of soft tissues. Of eight diagnoses of fracture with conventional imaging, two were classified as osteomyelitis and two as osteoarthritis using SPECT/CT. Overall, SPECT/CT findings led to revision of diagnostic category in 23 out of 71 patients (*p* < 0.01) [39] (Figure 6).

## 4. Evaluation of Pain of Spinal Origin

**Figure 7:**
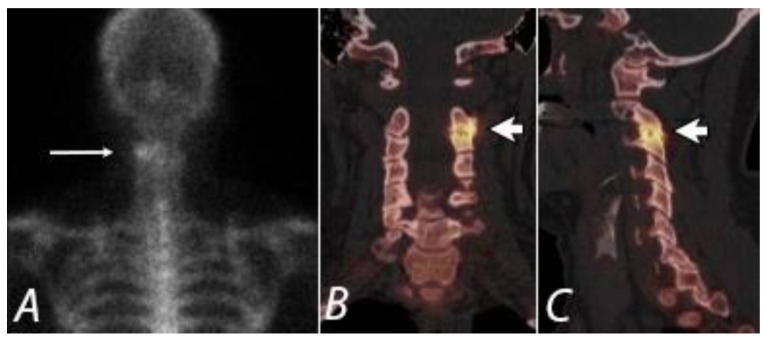
Posterior planar (**A**) (long white arrow), coronal (**B**), and sagittal (**C**) SPECT/CT images from a ^99m^Tc bisphosphonate bone scan of a 43 year old women with left neck pain. Marked radiopharmaceutical uptake corresponds to a severely degenerative left C2/3 facet joint (short white arrows), identifying a potentially targetable pain generator.

Chronic pain of spinal origin due to degenerative disease is a common encounter in clinical practice. Mechanical low back pain is a major health problem, which is associated with substantial economic and social costs [40] and accounts for up to 97% of low back pain diagnoses. It is mainly caused by degenerative disc disease, recurrent disc herniations, spondylolisthesis, degenerative scoliosis, or degenerative facet joint disease. Identification of the primary pain generator in these patients can be very difficult. Anatomic imaging modalities such as plain radiography, CT, or MRI can identify the sites of disc space degenerative and facet joint degenerative changes or other anatomic abnormalities but fail to accurately localize the exact sites of origin of pain. However, bone scintigraphy with SPECT or hybrid SPECT/CT imaging permits accurate localization of metabolically active sites of disease, thus aiding in appropriate management of these patients. ^99m^Tc-MDP bone SPECT has been shown to be predictive of active facet joint disease [41,42]. In a study by Ryan et al., 34 patients with chronic back pain were examined to determine the nature and clinical relevance of lesions identified on plain radiography, CT, or bone scintigraphy with SPECT. Of 27 patients with lesions identified on SPECT, 24 (89%) had abnormal CT findings and 18 (67%) had abnormalities on radiography. SPECT aided in the identification of 54 lesions, of which only 20 (37%) were detected with planar imaging. 43 SPECT lesions were located at the site of an abnormality also seen at CT and 20 at the site of an abnormality also seen at radiography. They concluded that bone SPECT provides diagnostic information that is not available with radiography or planar imaging, and the majority of the lesions seen at SPECT correspond to identifiable disease at CT. Few studies have demonstrated the utility of SPECT in localizing active facet joint disease and identifying the sites for targeted facet joint injections [42,43,44]. SPECT and SPECT/CT can guide in therapeutic intervention by differentiating facet joint arthropathy from costovertebral joint osteoarthritis [43,44] (Figure 7).

^99m^Tc bisphosphonate SPECT and SPECT/CT may be useful in patients with osteoporosis associated vertebral body collapse presenting with chronic back pain in whom the origin of pain could be facetal rather than related to fracture itself. Ryan et al. [45] has reported that 50% of the sites of increased uptake identified on SPECT were localized to facet joints. Facet uptake is more common in those patients with a higher degree of vertebral body collapse, and the facet joints immediately above or below the level of collapse were more commonly involved. The study concluded that a subgroup of patients with osteoporotic collapse with chronic back pain could benefit from treatment of these facet joints. It should be noted, however, that there are no head-to-head studies comparing SPECT/CT to MRI for this indication. Given the sensitivity of this modality for bone marrow edema and the lack of associated ionizing radiation, MRI will almost certainly remain the mainstay of imaging for back pain, with SPECT/CT reserved for problem-solving cases or for those patients in whom MR is contraindicated.

## 5. Evaluation of Postoperative Spine

The management of low back pain varies from conservative management to more invasive procedures such as spinal stabilization surgery that involves the placement of metallic screws, rods, plates, and other invasive procedures such as decompression, laminectomy, or discectomy. Indications for back surgery include spinal instability, degenerative disc disease, disc herniation, transfer lesions, and pseudoarthrosis [46,47,48,49]. Recurrent pain after spinal surgery is a well-known problem and is referred to as failed back syndrome and it is reported that up to 15–30% of patients experience persistent or recurrent pain after spinal instrumentation [50,51]. Patients can present with pain in the early post-surgical phase or later in the course of healing or re-modelling [52]. Few studies have estimated that the surgical reintervention rate is around 14% over a 4-year follow-up period and 19% over an 11-year period [53].

Accurate identification of patients who might benefit from reintervention is critical, as patient outcomes after reintervention surgery are relatively poor compared to initial surgery. Conventional imaging with plain radiography, MRI, or CT can evaluate for hardware position, alignment, hardware loosening, or possible non-union and pseudarthrosis. Though CT is very useful in identifying well-defined bony bridging, it is limited by low positive predictive value in the evaluation of evolving non-union or pseudarthrosis [54]. MRI evaluation of these patients is significantly limited due to metal artefact from the implants [55]. A number of studies have addressed the value of bone scintigraphy with SPECT/CT in the evaluation of failed back syndrome and concluded that the technique adds diagnostic value, which is helpful in patient management with better outcomes [49,56].

Pseudarthrosis or non-union, which leads to poor patient outcomes, is defined as an inability to form a well-defined osseous bridge, even after one year post surgery [52,57,58,59,60]. Physiologic re-modelling at the surgical site can demonstrate non-specific uptake, which is a major limitation of bone scans during the early postoperative phase. Gates et al. and Iseda et al. have reported the timeline of post-surgical physiologic uptake of tracer activity, with the highest uptake identified within a month post-surgery with gradual decrease of uptake starting around 3 months [61,62]. Persistent increased activity on ^99m^Tc bisphosphonate bone SPECT imaging beyond a year from surgery is considered as a possible sign of pseudarthrosis [63]. However, the pattern of uptake can aid in differentiating pathologic uptake from ongoing re-modelling at the surgical site—more focal uptake suggesting underlying pathology and a more diffuse uptake likely representing continuing remodeling. Even though SPECT is highly sensitive, it is limited by a significantly high false-positive rate of 42–50% [61]. Hybrid bone SPECT/CT has proven to be much more useful in the evaluation of pseudarthrosis due to inherent high sensitivity SPECT combined with the high specificity of CT and has been recommended by multiple studies as an effective modality in the evaluation of patients presenting with low back pain after spinal surgery [64,65,66]. The use of hybrid SPECT/CT as the imaging modality of choice has also been recommended for other causes of post-surgical complications after spinal surgery, including hardware failure, which could result from broken screws, misalignment, subsidence, and loosening [67,68,69,70].

## 6. Hip Pain

Planar ^99m^Tc bisphosphonate bone scintigraphy with or without SPECT was found to be useful in the evaluation of patients presenting with unexplained hip pain, especially if MRI is contraindicated or if findings on MRI are equivocal. However, with the advent of hybrid imaging, bone scintigraphy with SPECT/CT has become a comprehensive tool that not only enables whole-body skeletal assessment, in contrast to MRI, but also allows for detailed regional evaluation using targeted SPECT/CT acquisitions. Current indications for the use of SPECT/CT in the evaluation of painful hip include impingement, stress fractures or occult fractures, avascular necrosis, heterotopic ossification, assessment of joint replacements for loosening or infection, inflammatory or infective arthritis, or hip pain unexplained by other imaging modalities.

The use of SPECT/CT has been useful in diagnoses that may have otherwise been attributed to degenerative disease such as femoral acetabular impingement syndrome [71]. Femoral acetabular impingement syndrome is usually seen in young patients and is characterized by development of early osteoarthritis, which results from an anatomical structural abnormality of the acetabular cup or the femoral head [72,73]. Early identification of FAI as a cause of osteoarthritis and appropriate intervention can prevent development of early osteoarthritis in these patients, and MR arthrography is the preferred modality of choice for this purpose. SPECT/CT is an alternative modality of imaging in situations where MRI is not possible. Uptake in the superior femoral neck in association with osteoarthritic uptake of the superior hip joint on bone SPECT/CT has been reported to be suggestive of either cam or mixed cam–pincer impingement [74]. 

Diagnosis of stress fractures in patients on the chronic use of bisphosphonates for the treatment and prevention of osteoporosis can be subtle on anatomic imaging, but it can be easily identified on bone scintigraphy with SPECT/CT. These stress fractures are characteristically seen as involving the sub-trochanteric region or proximal femoral diaphysis [75].

Similar superior diagnostic properties were described for bone scintigraphy with SPECT/CT for conditions such as avascular necrosis, transient osteoporosis, and heterotopic ossification leading to hip pain [76].

Despite the tremendous success of total hip arthroplasty performed as a treatment for osteoarthritis of the hip, up to 25–30% patients present with persistent pain after the procedure [77,78]. The most common underlying etiologies for this persistent pain include loosening in approximately 10% of patients and infection in 1–2% of patients. The other causes implicated for the pain include instability, malposition, periprosthetic fractures, and heterotopic ossification [79]. Differentiation between aseptic loosening and infection is of paramount importance as the management of these patients differs in that aseptic loosening can be managed with one-stage definitive revision surgery, while infection requires two stage revision surgery [79]. 

Three phase ^99m^Tc bisphosphonate bone scan has been a widely used modality for evaluation and differentiation of the most common complications of loosening and infection. For up to 12 months after insertion of a hip prosthesis, periprosthetic uptake patterns are extremely variable, even around the asymptomatic devices due to remodeling, and it is usually advised to wait at least 1 year to perform bone scintigraphy after hip arthroplasty. While up to 10% of patients with cemented hip prosthesis will demonstrate uptake after one year, this is much more frequent with cement-less devices, and in this situation the uptake can be seen up to 2–3 years. Gelman et al. reported that bone scintigraphy was 85% accurate for prosthetic hip loosening and Weiss et al. reported that bone scintigraphy accurately identified prostheses requiring surgical intervention [80]. The uptake patterns of the radiotracers were evaluated to increase the specificity of the bone scan, with focal periprosthetic uptake suggesting loosening and diffuse uptake indicating possible infection [81]. Further, the diffuse pattern of uptake was found to be sensitive for infection and not specific according to a study published by Williams et al. [82]. Blood pool phase images were found to be valuable in assisting in the differentiation of loosening from infection, with lack of pool activity suggesting an absence of infection. 

Triple phase ^99m^Tc bisphosphonate bone scan with the addition of SPECT/CT increases the specificity and diagnostic accuracy of scintigraphy with the evaluation of bone and soft tissue morphology with the CT component. The CT portion of the study can provide additional information such as adjacent fluid collections and fistulous tracks, which suggests infection as a possible cause of implant failure rather than aseptic loosening. Strobel et al. and Tam et al. have reported that SPECT/CT with triple phase bone scan is a promising tool in the evaluation of post total hip arthroplasty complications. In a study by Arican et al., the reported sensitivity for triple phase bone scan was 77% and 93.7% for bone SPECT/CT in the evaluation of post hip arthroplasty complications [83,84]. 

One exception to waiting for one year to perform a bone scan is if there is concern for heterotopic ossification as this condition affects the adjacent soft tissues where no uptake is expected. Bone scan along with SPECT/CT not only helps in identifying this soft tissue uptake, but also helps in the extent and activity of the disease, which helps in the timely planning of surgical intervention [85,86].

## 7. Knee Pain

MRI, with its ability to evaluate the soft tissue structures such as menisci, ligaments, cartilage, and synovium apart from the osseous structures, is the imaging modality of choice in the evaluation of patients presenting with knee pain. MRI is also valuable in characterization of neoplastic processes around the knee joint and infective/inflammatory arthritis. However, MRI is of limited value in the presence of metallic implants due to susceptibility artefact, even with the development of metal artefact reducing MRI sequences and also in the evaluation of cortical bone [87]. ^99m^Tc bisphosphonate scintigraphy with SPECT/CT has been shown to be superior in these situations as well as in other situations where MRI is contraindicated due to the presence of pacemakers or metallic foreign bodies and in patients with renal insufficiency. 

**Figure 8:**
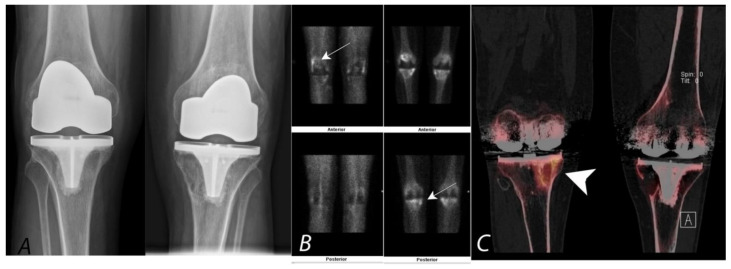
Bilateral anterior AP radiographs, anterior and posterior planar blood pool and delayed mineral phase images and coronal SPECT/CT images of a Tc-99m MDP bone scan in a 65-year-old male with bilateral painful total knee protheses. Plain radiographs (**A**) are non-specific (and unchanged over several years) without defined peri-prosthetic lucency. Blood pool images and delayed images (**B**,**C**) demonstrate some hyperemia in the joint capsule and serpiginous areas of proximal tibial uptake (white arrows). Clarity is achieved by the SPECT/CT (coronal images, (**C**)), which demonstrates uptake associated with large, well-corticated areas of bone loss consistent with particle disease (white arrowhead). A similar well corticated region of lucency is also present in the medial aspect of the left proximal tibia.

SPECT/CT, along with knee arthrography, can be used to evaluate soft tissue structures such as cartilage, menisci, and ligaments. This technique has been shown to be useful in the assessment of meniscal tears and injury to the ligaments, as well as in identifying intraarticular loose bodies within the knee joint [88,89,90]. SPECT/CT has also been shown to be useful in the evaluation of tumors around the knee joint and osteochondral defects and assessing remodeling at the sites of ligamentous repairs, as well as in the evaluation of patients presenting with pain after total knee arthroplasty [91] (Figure 8).

**Figure 9:**
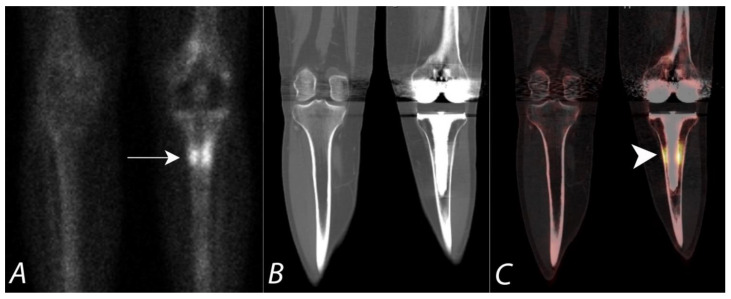
Fifty-year-old female with a painful left knee prothesis. Anterior planar (**A**), coronal CT (**B**) and coronal SPECT/CT (**C**) images of a Tc-99m MDP bone scan of both knees demonstrate a prominent increased uptake in a band surrounding the midportion of the left tibial prosthetic stem. Plain AP radiographs demonstrate sclerosis without periprosthetic lucency. Findings are consistent with being related to altered biomechanical remodeling (similar to a stress fracture). There is no evidence for loosening or infection.

SPECT/CT has become a very useful technique in the evaluation of painful knee status post knee arthroplasty and has recently been proposed as the second line of imaging modality after MRI in these patients. Even in asymptomatic patients, radiotracer uptake on planar and SPECT bone scans, specifically around the tibial component of the arthroplasty, can be a normal finding, even many years after the procedure. The addition of the CT component to SPECT significantly increases the specificity and accuracy of diagnostic interpretation by improving accurate localization of the uptake to a specific anatomic location [92,93]. For example, identification of the granulomatous process on CT can be a cause for non-specific diffuse uptake on bone scans. Additionally, in the absence of any anatomic abnormalities on CT, localized radiotracer activity below the medial aspect of the tibial component can be interpreted as physiological with a greater degree of confidence. The addition of other follow-up evaluation procedures, specifically if infection is suspected, may be required for complete assessment of painful knee after arthroplasty. SPECT/CT has been shown to be a highly valuable modality in this subset of patients, with alteration of management approach in up to 85% patients [92,93] (Figure 9).

## 8. SPECT/CT of Extremities

**Figure 10:**
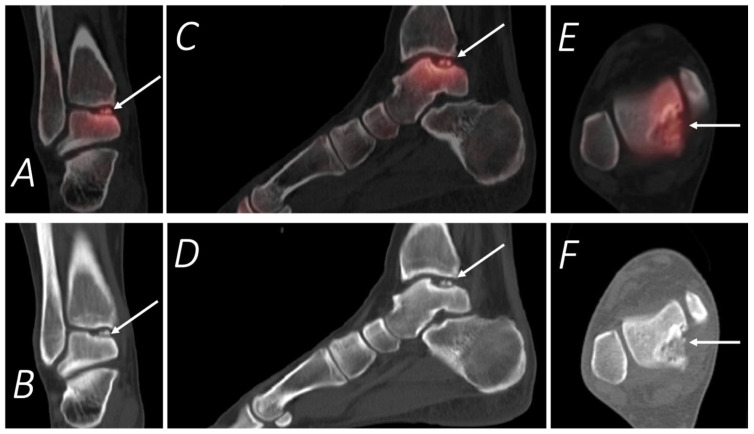
Thirty-two-year-old female being evaluated for chronic but worsening right ankle pain underwent a Tc-99m MDP bone scan with SPECT/CT. Selected upper panel SPECT/CT and corresponding lower panel CT images of the right ankle in coronal (**A**,**B**), sagittal (**C**,**D**), and axial (**E**,**F**) projections are provided. The white arrow denotes a defect in the posteromedial talar articular cartilage and underlying subchondral bone, which corresponded symptomatically with the region of the patient’s pain.

The complex architecture of bony articulations involving the small joints of wrist, ankle, and foot make evaluation of these structures difficult on planar or SPECT imaging without anatomic landmarks for accurate localization of tracer activity. Most often, any uptake that is identified localizing to these joints on planar or SPECT images is attributed to degenerative changes. However, SPECT/CT demonstrates the specific anatomy corresponding to a focus of uptake. This added advantage often enables the identification underlying causes of uptake, allowing the interpreter to distinguish between degenerative change, tumor, and occult trauma. Mohan et al. reported that the addition of SPECT/CT in patients presenting with ankle/foot pain has provided additional diagnostic information in 81% of patients and aided in changing the management plan in up to 62% of patients [94]. A comparison study was performed using SPECT/CT with MRI versus MRI alone on 25 patients with osteochondral defects involving talus. Stand-alone SPECT/CT interpretation has changed the management approach in 48% of patients, whereas SPECT/CT combined with MRI interpretation changed the treatment decisions in 52% of patients [95]. In an additional study, a significant correlation was reported between positive SPECT/CT studies with increased focal activity localizing to an osteochondral defect involving the ankle joint and significant relief of pain after local anesthetic infiltration into the ankle joint [96] (Figure 10).

**Figure 11:**
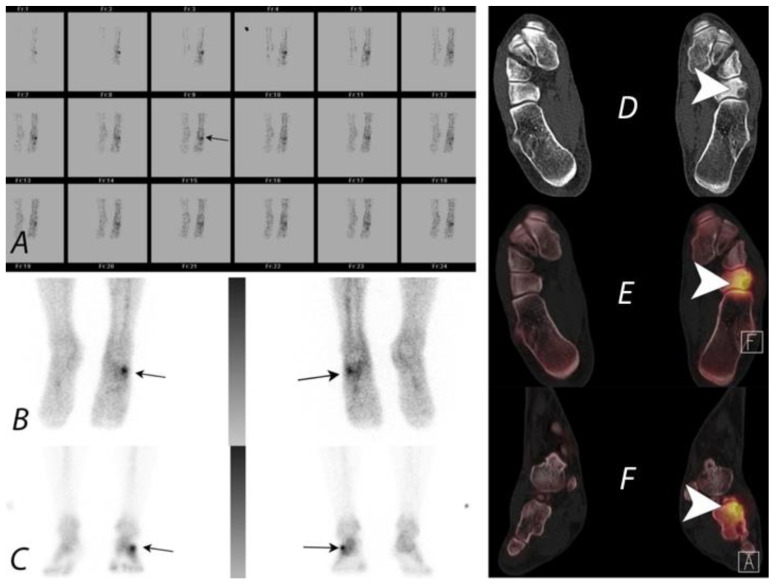
A young patient presenting with chronic left foot pain underwent three phase bone scan with SPECT/CT. Radiographs were interpreted as normal (not shown). Blood flow and pool images (**A**,**B**) demonstrate increased blood flow and pool activity with focal accumulation of activity on the delayed images (**C**) along the lateral aspect of the mid foot (black arrows). Axial CT image (**D**) and fused axial (**E**) and fused coronal (**F**) SPECT/CT images localize the activity to a well-defined lytic lesion with (white arrowheads) well defined sclerotic rim and a nidus involving the cuboid bone and was interpreted as a possible osteoid osteoma. Patient underwent biopsy, which revealed the lesion to be a benign cartilaginous neoplasm.

SPECT/CT of the foot and ankle has also been shown to be valuable in the assessment of post joint fusion, tarsal coalition, post-operative infection, Achilles tendonitis, and plantar fasciitis, along with stress fracture and painful accessory bone syndromes [94,95,96,97,98,99]. Even though anatomic imaging modalities such as ultrasonography and MRI may remain the initial imaging modality of choice for these clinical scenarios, SPECT/CT can provide additional diagnostic information. The CT portion of the SPECT/CT can provide for more focused assessment of areas of increased radiopharmaceutical uptake in patients presenting with continuing pain after joint fusion with hardware in place, and aid in diagnosing specific causes such as non-union/mal-union, loosening, or degenerative change as the cause of the pain [94]. Evaluation of patients with tendinitis of the Achilles tendon and other similar pathologies is limited on planar bone scans. However, these patients can benefit from the use of SPECT/CT, which provides additional useful information and helps the clinician in better patient management [94]. The CT portion of the SPECT/CT can identify soft tissue abnormalities involving the bursae, fat pad abnormalities as well as in the evaluation of occult or stress fractures. SPECT/CT has been shown to be useful in the evaluation of tarsal coalition and painful accessory bone syndromes and unexplained foot pain (Figure 11), although no studies to date have compared this technique with CT alone or MR imaging. SPECT/CT is particularly useful in the assessment of foot and ankle pathology after surgery when imaging with CT, and MRI may be limited by metallic artefact [94].

## 9. Infection and Inflammation

With improved tissue contrast and precise anatomic localization of radionuclide deposition and superior definition of bone morphology, SPECT/CT offers many advantages over planar or SPECT only imaging for skeletal infection or inflammation. Identification of bone infection and the distinction between osteomyelitis and other bone pathology creates challenges in diagnostic imaging. In meeting these challenges, a number of imaging strategies have been developed. Initial imaging is typically performed by plain radiography and CT, which may reveal destructive changes of the bone, periosteal reaction, sequestra, and involucra in advanced cases of osteomyelitis. However, early stages of osteomyelitis may be difficult to recognize by conventional X-ray imaging methods. 

Contrast-enhanced MRI is often the first advanced imaging method performed in the case of suspected osteomyelitis. MRI imaging may identify hyperemia and edema in the bone in osteomyelitis, associated soft tissue sites of infection, fluid collections such as abscesses, and complications such as necrotizing fasciitis. MRI also offers superior anatomic definition. However, the presence of hardware at the site of concern, ferromagnetic metal in the body, an inability to administer contrast, severe peripheral vascular insufficiency, heterogenous distribution of bone marrow, and patient intolerance may limit utilization and diagnostic utility of MRI in some cases. Furthermore, MRI and conventional X-ray techniques may be inaccurate in distinguishing between Charcot changes and osteomyelitis in the diabetic or neuropathic foot or joint. Recent developments to lift CMS restrictions on the use of ^18^F-fluorodeoxyglucose (FDG) PET-CT for infection was followed by exclusion of coverage for chronic osteomyelitis, fever of unknown origin, and the infected hip protheses [99]. Although the negative predictive value is high, the FDG PET is limited in specificity in that non-specific inflammatory pathologies may show increased uptake on FDG PET. Therefore, the future of insurance or Medicare reimbursement of FDG PET for bone infection is uncertain. 

**Figure 12:**
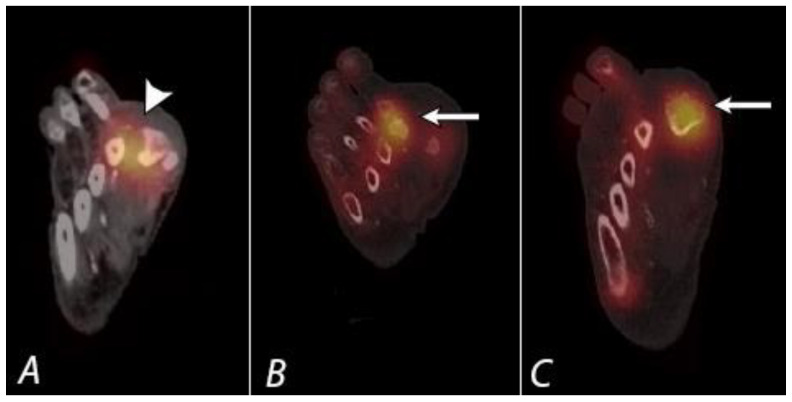
Fifty-eight-year-old diabetic male with history of right great toe amputation (at the MTP joint) 2 months previously, now with new pain, swelling, and redness in the medial forefoot. ^99m^Tc MDP SPECT/CT bone scan (**B**,**C**) shows increased uptake at the first metatarsal head and the second MTP joint (white arrows). An ^111^In WBC SPECT/CT scan (**A**) shows concordant uptake as well as a larger area of soft tissue uptake around first metatarsal head and the second MTP joint, confirming cellulitis in addition to osteomyelitis (white arrowhead).

Diagnostic imaging using radionuclides offer a number of tools for identifying osteomyelitis and distinguishing it from other pathologic conditions. A conventional three-phase bisphosphonate bone scan for identification of osteomyelitis relies upon the identification of marked focal hyperemia on angiographic and equilibrium blood pool imaging and corresponding focal increased uptake on the delayed (mineral phase) images. A number of bone pathologies may be associated with various degrees of hyperemia, including non-pyogenic acute or chronic inflammatory lesions, actively forming heterotopic bone, tumors, and trauma. An absence of hyperemia on the three-phase bone scan is strongly predictive of the absence of infection, providing that the patient does not have severe peripheral vascular insufficiency to limit the hyperemic response to infection. The use of SPECT/CT has been shown to add significant value when used with the three-phase bone scan [100]. SPECT/CT can often distinguish between the differential considerations of a positive scan by identifying specific morphologic features of the bone at the site of radionuclide uptake. In addition to the three-phase bisphosphonate bone scan, additional radionuclide strategies to use in patients with suspected bone infection are the leukocyte, or white blood cell (WBC) scan, labeled with either ^99m^Tc HMPAO or ^111^In oxine. WBC scans that employ SPECT/CT have been shown to be of significant value in distinguishing soft tissue from bone infection [101]. WBC SPECT/CT can also be used in combination with Tc-99m bisphosphonate SPECT/CT for added confirmation of osseous remodeling (Figure 12).

**Figure 13:**
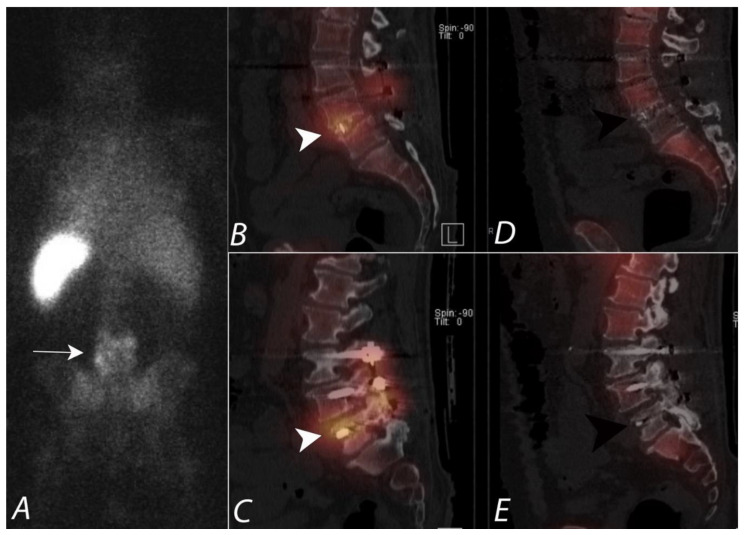
Seventy-two-year-old male with draining posterior lower lumbar wound one month after dorsal decompression and bilateral dorsal fixation from L3 to S1. Posterior planar image from an ^111^In leukocyte (WBC) scan (**A**) demonstrates heterogeneous uptake of tracer activity localizing to the lower lumbar region (white arrow). Sagittal SPECT/CT images of the ^111^In WBC scan (**B**,**C**) show increased uptake of labeled WBCs in the soft tissues, posterior elements, disc space, and vertebral bodies at L4-5. (white arrowheads). However, sagittal SPECT/CT images from ^99m^Tc sulfur colloid scan (**D**,**E**) demonstrate no focal uptake (black arrowheads) and is incongruent with the ^111^In WBC distribution of activity. This supports that a pyogenic infection is present involving the L4-5 vertebrae, posterior elements, and disc spaces. The absence of uptake on sulfur colloid confirms that the uptake of WBCs is not due to a granulation tissue due to mononuclear cell infiltration in chronic inflammation.

In a number of published reports, SPECT/CT with labeled WBCs and Ga-67 scans have been shown to provide useful information, providing improved distinction between uptake in soft tissues vs bones, defining the extent of abnormality and improving specificity and diagnostic certainty [102,103]. In the case of WBC scans, sensitivity for peripheral bone infections is very high but sensitivity for central osteomyelitis (in vertebrae or red-marrow containing portions of the skeleton) is much lower, due to early devascularization of infected vertebrae (preventing influx of labeled WBCs) or a similar degree of uptake of labeled WBC in vertebral osteomyelitis to that in normal red marrow [104]. In the latter circumstance, a comparison between the WBC scan and a companion ^99m^Tc sulfur colloid scan (which shows normal marrow) can be helpful in identifying subtle vertebral osteomyelitis. The improved anatomic definition provided by SPECT/CT is important in this application. In the spine, osteomyelitis will result in replacement of the normal red marrow with neutrophils. The typical osteomyelitis in red marrow containing portions of the skeleton shows uptake of labeled WBCs, which may be similar to background red marrow, but not of ^99m^Tc sulfur colloid (Figure 13). Unfortunately, 25% of cases of spinal osteomyelitis will be “cold” on the WBC scan. This is likely due to devascularization of the vertebral body, resulting in poorly penetrated by the WBCs. In addition, chronic osteomyelitis may be poorly visualized on labeled WBC scans, in theory, due to the “walling off” of the infection.

**Figure 14:**
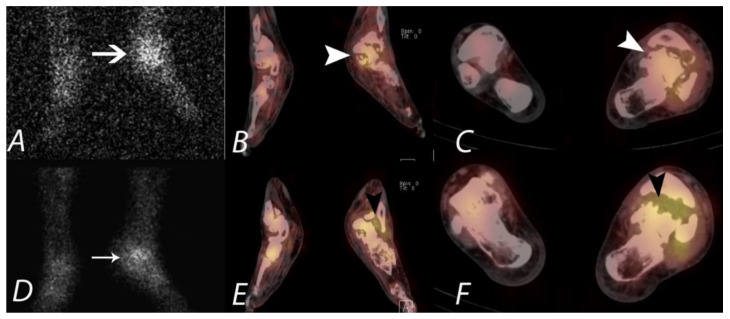
A seventy-year-old diabetic male patient with chronic left midfoot Charcot joint changes was evaluated for possible superimposed infection. Planar image (**A**), coronal fused axial image (**B**), and axial fused axial SPECT/CT image (**C**) from In-111 WBC scan demonstrate focal uptake localizing to mid and hind foot (large white arrow and white arrowheads), which is congruent with uptake identified on Tc-99m sulfur colloid images (**D**–**F**), supporting the presence of chronic inflammation and granulation tissue and not active infection (small arrow and black arrowheads).

WBC scans are also useful in the distinction between bone infection as well as associated soft tissue infection, for which SPECT/CT is critical. False positive WBC scans can be seen with hemorrhage, trauma, or chronic non-pyogenic inflammatory conditions, particularly Charcot joints in diabetics. In the case of Charcot joints, chronic neuropathic injury and bleeding results in a chronic inflammatory condition with extensive granulation tissue. This tissue can result in uptake of labeled WBCs, even in the absence of infection. Concurrent or sequential imaging using both labeled WBC and ^99m^Tc sulfur colloid scans is a method to distinguish chronic non-infected inflammation from infection, particularly in the Charcot foot and in portions of the skeleton containing red marrow [105,106]. In labeling WBCs, a preparation that is composed of a heterogenous collection of WBCs as well as RBCs and platelets is utilized. As such, monocytes are also labeled. In chronic inflammation, for example Charcot joints, monocytes localize at the site and differentiate into macrophages, which are phagocytic. Labeled WBCs also localize to the normal red marrow. These phagocytic cells engulf ^99m^Tc sulfur colloid particles when injected intravenously. Therefore, concordance between the uptake on the labeled WBC scan and the ^99m^Tc sulfur colloid is indicative of chronic inflammation, such as is seen with a Charcot joint or with normal red marrow, but not with pyogenic infection (Figure 14). With infection, neutrophils rapidly replace all other cell types, including granulation tissue, resulting in discordance between labeled WBC and ^99m^Tc sulfur colloid scans. SPECT/CT offers better anatomic comparison of the distribution of labeled WBC and ^99m^Tc sulfur colloid. Rigorous immobilization during SPECT/CT imaging is critical to insure accurate comparisons.

Historically, ^67^Ga citrate has been used as an alternate method to diagnose chronic or other osteomyelitis. Although normal bone uptake of ^67^Ga occurs by chemisorption onto the mineral component of the bone, uptake of ^67^Ga in infection occurs by binding to lactoferrin deposited by degranulation of neutrophils [107]. ^67^Ga also binds to bacterial siderophores at the site of infection. When ^67^Ga citrate is utilized for assessment of bone infection, it is important to perform a companion ^99m^Tc bisphosphonate bone scan, and comparison is made between the magnitude of uptake in adjacent normal bone to the shape and magnitude of uptake in the bone region of concern. It is typically best to perform the bone scan first, then inject with 5 mCi ^67^Ga citrate and perform imaging at 48–72 h. Some advocate that imaging at 24 h can be carried out, but this results in poorer bone localization. When uptake of ^67^Ga is greater (compared to surrounding normal bone) than in is uptake on the ^99m^Tc bisphosphonate bone scan, or if it is similar in uptake but different in shape, the study is considered positive for active osteomyelitis. If the uptake on ^67^Ga in the lesion is less, compared to normal bone, than is the uptake on the bone scan, the study is considered negative for osteomyelitis. When uptake on the two scans is similar, then the study is non-diagnostic [108]. The positive and negative predictive values are both excellent for the dual tracer study. However, 72% of cases of confirmed osteomyelitis are non-diagnostic, reducing the overall value of this approach. For this reason, a labeled WBC scan, typically with a companion ^99m^Tc sulfur colloid scan, is typically preferred over ^67^Ga for questions of osteomyelitis.

**Figure 15:**
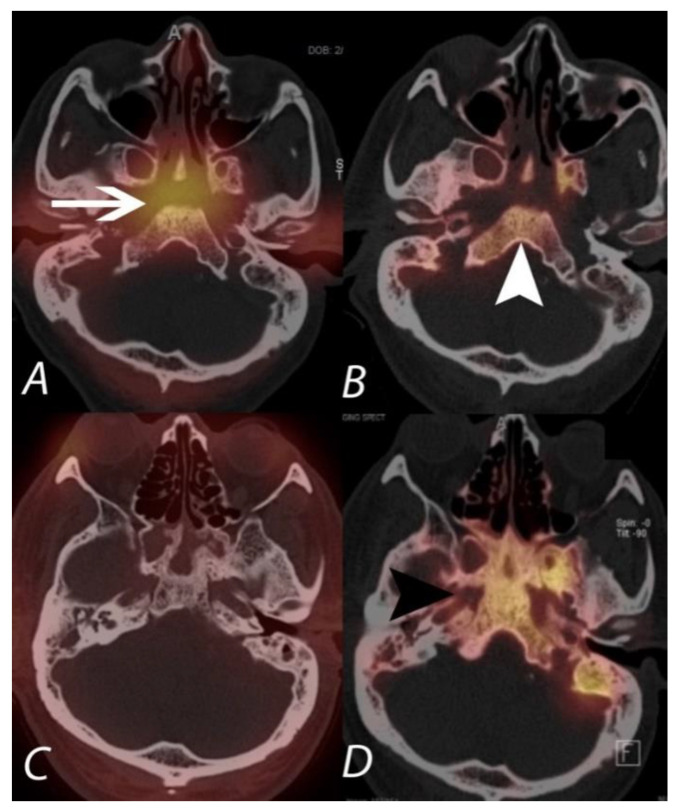
The upper panel (**A**,**B**) demonstrates ^67^Ga and ^99m^Tc MDP SPECT/CT scans in a patient being evaluated for acute malignant otitis externa. While ^99m^Tc MPD SPECT/CT shows uptake in the bones of the central skull base (white arrowhead), consistent with osteomyelitis, the ^67^Ga also demonstrates soft tissue infection at the central skull base and around the external auditory canals. The lower panel (**C**,**D**) is of a different patient with continued pain following completion of an antibiotic regimen for skull base osteomyelitis. The ^99m^Tc MDP SPECT/CT (right lower panel) shows post-infectious remodeling hyperostosis of the bones of the central skull base, but the ^67^Ga SPECT/CT (left lower panel) shows an absence of active infection.

The one instance in which ^67^Ga scanning is still frequently used in preference to other imaging options is in combination with a ^99m^Tc bisphosphonate bone scan for the assessment of active external malignant otitis or other skull base osteomyelitis [109]. In this case, SPECT/CT is critical for detailed definition of sites of uptake, which cannot be adequately defined by planar imaging. The ^99m^Tc bisphosphonate bone scan is typically carried out first, with SPECT/CT as an essential part of this study. The patient is then injected with ^67^Ga and repeat SPECT/CT is carried out at 48–72 h. ^67^Ga can not only reveal site of active infection in the bone, but also associated soft tissue infection (Figure 15). A similar strategy to that described above is applied to identify active infection as a relatively increased magnitude or distribution of ^67^Ga uptake compared to that of the ^99m^Tc bisphosphate bone scan. In summary, the addition of SPECT/CT has been shown to raise diagnostic confidence, improve specificity, and provide significant clinical contribution in the diagnosis of musculoskeletal infection.

## 10. Extraosseous Uptake of Tracer on Bone Scans

A myriad of non-osseous processes can demonstrate soft tissue uptake of ^99m^Tc-MDP, including neoplastic, hormonal, inflammatory, ischemic, and traumatic entities, but they can also be related to technical factors. Extraosseous uptake is usually incidental and unexpected, and it is important to exclude technical and quality control causes as these represent the majority of cases. It is also important to obtain a thorough clinical history, especially with regards to any recent other nuclear medicine examinations that the patient could have undergone in the past few days. Various mechanisms implicated in extra-osseous uptake include enhanced regional vascularity and permeability, extracellular fluid expansion, altered calcium metabolism with increased tissue content of calcium, and binding to denatured proteins, among other causes [110,111].

**Figure 16:**
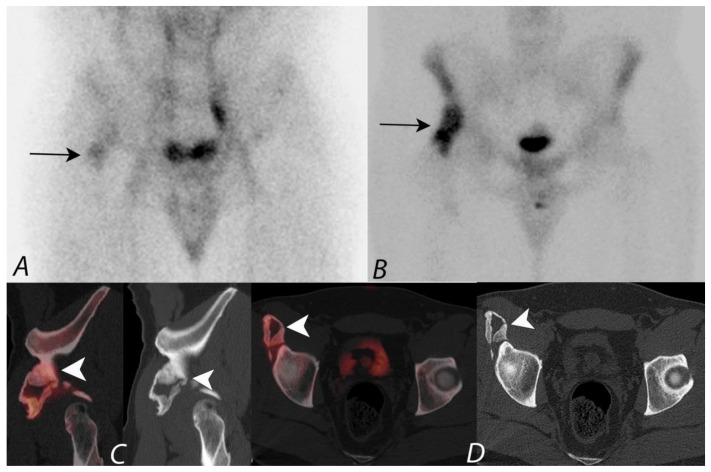
Patient presenting with right hip and with clinical history of possible heterotopic ossification. Planar blood pool image (**A**) and delayed image (**B**) in anterior projection from a ^99m^Tc MDP bone scan demonstrating increased blood pool activity and delayed focal uptake localizing to the right hip region (long black arrows). Fused sagittal image with correlative CT image (**C**) and fused coronal with correlative CT image (**D**) localizes the focal activity to an area of exuberant heterotopic ossification along the anterior aspect of the anterior wall of right acetabulum. Given increased blood pool activity, the heterotopic ossification is immature and represents an ongoing process.

The angiographic phase of the bone scan reflects relative regional blood flow. During the blood pool or soft tissue phase of the three phase bone scan, the radiopharmaceutical rapidly enters the extra-cellular space [112]. The characteristic distribution of the tracer activity on delayed images is related to the binding of the radiopharmaceutical to hydroxyapatite crystals and calcium salts. Intact preserved regional blood flow is a prerequisite for radiotracer delivery and uptake in soft tissues and bone. An absence of blood flow in pathologic conditions such as frostbite or acute avascular necrosis explains the lack of radiotracer uptake in these situations [110,113,114]. Hybrid SPECT/CT has been shown to improve diagnostic interpretation by increasing specificity and accurate localization of radiotracer accumulation [115]. An understanding of the physiologic and pathophysiologic mechanisms of radiopharmaceutical uptake is critical in determining the underlying cause [116]. Once technical/quality control factors are excluded as a cause, then the encountered extra-osseous radioactivity may be related to altered bio-distribution [110,115,117]. Multiple studies have reported the usefulness of SPECT/CT in the evaluation of heterotopic ossification, as accurate localization of activity is limited on planar imaging. The accumulation of radioactivity in this situation often overlaps the adjacent osseous structures. However, SPECT/CT allows accurate localization of the activity within the adjacent/underlying osseous structures as well as confirming the abnormal uptake within the adjacent soft tissues related to heterotopic ossification [118,119] (Figure 16). It has also been reported that the use of SPECT/CT can accurately diagnose heterotopic ossification and differentiate from clinically suspected osteomyelitis [120]. 

## 11. Frostbite

Frostbite results from localized cold-induced tissue injury from prolonged exposure to freezing or near freezing temperatures, and it involves the hands and feet in more than 90% of cases. Frostbite is frequently seen in places with extreme environments or industrial settings from accidental exposure to refrigerant chemicals or dry ice, as well as in households from misuse of cold packs or fire extinguishers. Frostbite has a predilection for those who are unable to protect themselves from adverse environmental situations such as wilderness hikers, substance abusers, psychiatric patients and alcoholics, as well as in patients with coexisting conditions such as diabetes mellitus. The severity of frostbite depends on the duration of exposure to the cold thermal insult. 

**Figure 17:**
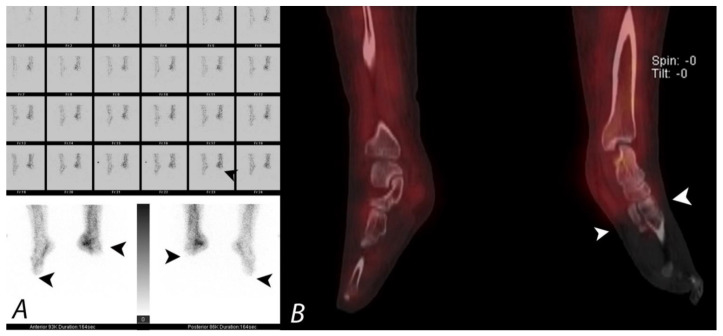
Patient presenting with severe cold injury (frostbite) to bilateral feet underwent three phase bone to evaluate for the extent of devascularization as a part of presurgical evaluation. Blood flow images and blood pool images (**A**) demonstrate absent perfusion and pool activity involving the fore foot and mid foot on the left side and involving the forefoot on the right side. Coronal SPECT/CT (**B**) confirms the findings with accurate delineation and demarcation of viable and non-viable tissue. Patient underwent bilateral below knee amputation.

Scintigraphic perfusion imaging with angiographic and equilibrium blood pool imaging, using either ^99m^Tc bisphosphonate or pyrophosphate, plays an important role in the evaluation of frostbite or other devascularizing injuries. This technique can inform clinical management by determining the level of soft-tissue non-perfusion to facilitate amputation planning or attempted reperfusion techniques. The expedited determination of this information can have major implications on patient management in preventing the potential catastrophic development of gangrene. In the case of frostbite, attempted intravascular reperfusion must be performed expeditiously, so scintigraphic perfusion imaging should be performed early and can also be used to determine success in reperfusion. Perfusion imaging is also important in surgical management with amputation aimed at a level that will allow the stump to be well-covered by well-perfused soft tissues. SPECT/CT obtained during the blood pool phase allows a precise definition of tissue perfusion relative to anatomic landmarks. (Figure 17). Because bone perfusion may not follow that of soft tissue perfusion, obtaining SPECT/CT images during the delayed phase may be misguiding. 

The patterns of uptake on each phase of the bone scan correlates well with the clinical management, with minor injury demonstrating hyper-perfusion and normal soft tissue and delayed uptake, requiring no surgical intervention on any part of the spectrum of severe injury, with deep gangrene and bone infarction requiring amputation and bone scan demonstrating absent uptake on all three phases [121].

## 12. Conclusions

SPECT/CT is a fascinating hybrid imaging modality that combines the functional information of scintigraphic studies with anatomic information. It has been shown in the literature that SPECT/CT improves diagnostic accuracy and increases confidence in interpretation of various nuclear medicine studies, with a significant impact on patient management and outcomes. The evidence base regarding the application of SPECT/CT in the evaluation of patients with suspected musculoskeletal pathology is increasing, with multiple studies demonstrating promising results in cancer patients and in the evaluation of benign musculoskeletal conditions. Although the use of ionizing radiation in SPECT/CT remains a limitation, improved specificity, accurate localization of abnormal uptake, characterization of the cause of uptake, and a consequent reduction of equivocal interpretations should be considered in clinical practice for increased adoption of this hybrid imaging modality.
